# Cardiac Angiosarcoma in the Right Atrium Treated by Surgical Resection

**DOI:** 10.3390/medicina60081321

**Published:** 2024-08-15

**Authors:** Milica Dragicevic-Antonic, Ljiljana Rankovic-Nicic, Gordana Stamenkovic, Masa Petrovic, Goran Loncar, Nikola Markovic, Ana Dimitrijevic, Sulin Bulatovic, Milan Cirkovic, Branislava Borzanovic, Zelimir Antonic, Maja Pirnat, Robert Manka, Milovan Bojic

**Affiliations:** 1 Institute for Cardiovascular Diseases “Dedinje”, 11000 Belgrade, Serbia; 2 Faculty of Medicine, University of Belgrade, 11000 Belgrade, Serbia; 3 Maribor University Clinical Center, 2000 Maribor, Slovenia; 4 University Hospital of Zürich, 8091 Zürich, Switzerland

**Keywords:** angiosarcoma, Takotsubo cardiomyopathy, cardiac magnetic resonance imaging, multidisciplinary approach

## Abstract

We present the case of a 49-year-old female of Caucasian European descent with chest tightness, fatigue, and palpitations, ultimately diagnosed with primary intracardiac angiosarcoma. Initial echocardiography revealed a significant mass within the right atrium, infiltrating the free wall. Surgical intervention included tumor excision and partial resection of the superior vena cava. Histopathological examination confirmed a high-grade angiosarcoma. Postoperative imaging identified a recurrent mass in the right atrium, suggestive of thrombus, alongside Takotsubo cardiomyopathy. Considering the elevated surgical risks and the presence of cardiomyopathy, management included anticoagulation therapy with Warfarin and adjuvant chemotherapy with Paclitaxel. Follow-up cardiac magnetic resonance imaging demonstrated a recurrent angiosarcoma with superimposed thrombus. This case presents the complex diagnostic and therapeutic landscape of angiosarcoma, highlighting the critical importance of early surgical intervention, advanced imaging techniques, and vigilant postoperative monitoring.

## 1. Introduction

Angiosarcoma (AS) represents the most prevalent primary malignant cardiac tumor, predominantly localized in the right atrium (RA) near the atrioventricular sulcus [1]. Originating from vascular endothelial cells, AS is marked by a high propensity for metastasis and local recurrence [2]. The identification of hemorrhagic and irregular masses within the RA is strongly indicative of primary cardiac angiosarcoma, even in the absence of other malignancies [3].

The prognosis for angiosarcoma remains poor due to its anatomical position and metastatic potential, frequently associated with hemorrhagic pericardial or pleural effusions [1,4,5]. AS is known for its aggressive infiltration of the myocardial wall, cardiac chambers, adjacent valves, vascular structures, and pericardium [6]. The clinical manifestations of AS are highly variable and contingent upon the tumor’s location, size, and degree of invasiveness. Dyspnea is the most prevalent symptom, while pleuritic chest pain is reported in approximately half of the patients diagnosed with angiosarcoma [7]. Right-sided AS typically presents as bulky, infiltrative masses with nonspecific symptoms, whereas left-sided AS primarily manifests as dyspnea due to local obstruction and resultant heart failure [8]. Furthermore, right-sided heart failure may ensue secondary to hemorrhagic pericardial tamponade or obstruction of the superior vena cava [9].

Echocardiography (ECHO) is a cost-effective and widely accessible imaging modality, serving as the initial method for detecting and evaluating cardiac masses [10]. Transesophageal echocardiography (TEE) provides detailed insights into the characteristics of these masses [10]. Advanced imaging techniques, such as computed tomography (CT) and cardiac magnetic resonance (CMR), offer comprehensive information regarding tumor localization, extension, and the characteristics of surrounding tissues [11]. CMR is particularly advantageous for its capability to provide tissue characterization, enabling the differentiation between pseudomasses and true masses, as well as benign and malignant lesions [1]. Despite the inherent cellular heterogeneity of AS, immunohistochemical markers should be interpreted in conjunction with other diagnostic findings, with endothelial origin tumors typically indicated by CD31, CD34, ERG, and factor VIII [12].

Surgical extirpation remains the treatment modality of choice for AS. Consequently, early diagnosis is imperative, and a multidisciplinary approach is essential to optimize patient outcomes [13].

## 2. Case Presentation

We present the case of a 49-year-old female of Caucasian European descent who was admitted with complaints of chest tightness, fatigue during moderate physical activity, and occasional sensation of palpitations occurring at rest and spontaneously resolving after a few seconds. The patient has a history of hyperthyroidism, hypertension, and type II diabetes mellitus treated with oral therapy (metformin 1000 mg twice daily), and an allergy to penicillin. The patient does not report any significant family history of malignant diseases.

**Figure 1:**
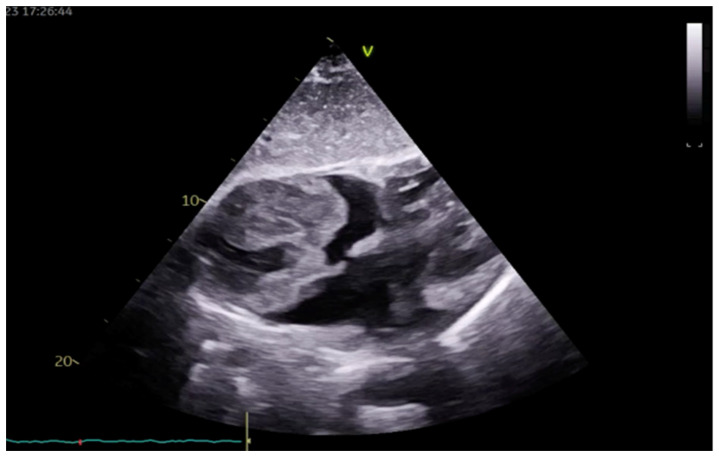
TTE subcostal view showing bulky and irregular mass occupying two-thirds of the RA.

Upon admission, ECHO revealed fibriotically altered mitral leaflets and minimal mitral regurgitation in the left atrium, which was normal in size. Examination showed that the left ventricle was of normal size, with globally preserved systolic function and no segmental wall motion abnormalities. Additionally, the ECHO revealed an enlarged right ventricle (RV) exhibiting moderately impaired RV function and a large mass in the RA without extension into the caval veins, infiltrating the free wall of the RA (Figure 1). The large echogenic mass was seen filling the entire RA, with varying echo densities and a cauliflower-like structure with smaller echo masses on the surface with increased embolic potential. Furthermore, the tricuspid valve leaflets were slightly fibrotically altered, appearing normal in mobility with mild regurgitation. An electrocardiogram (ECG) was normal, with a heart rate of 92 beats per minute without any ST- or T-wave abnormalities.

Based on these findings, the Heart team recommended surgical excision of the tumor mass from the RA, and then further decisions on systemic treatment would be made upon obtaining the pathohistological results. Preoperatively, CT imaging was performed to exclude pulmonary thromboembolism and any metastatic lesions, as well as to evaluate for pulmonary and abdominal lesions. Despite the metastatic nature of the diseases, there were no metastatic lesions on CT imaging. Coronary angiography excluded any significant stenosis of the coronary arteries. A Doppler of the carotid arteries revealed 25% bilateral stenosis.

**Figure 2:**
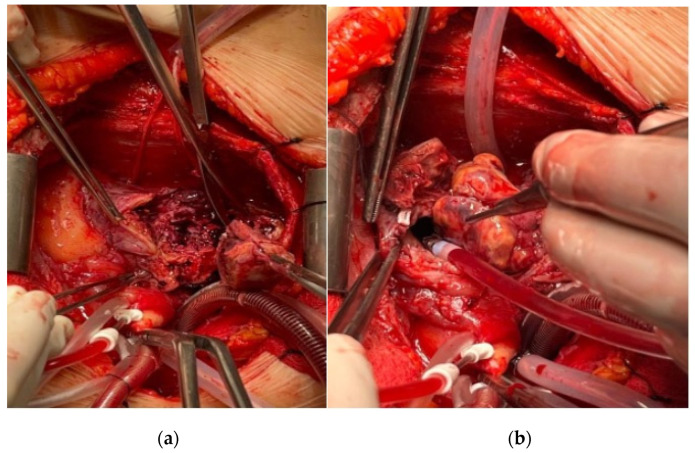
(**a**,**b**) Intraoperative findings during extirpation of angiosarcoma from RA and reconstruction with CorMatrix patch.

The patient subsequently underwent extensive tumor resection and partial resection of the superior vena cava, with reconstruction of the RA wall using a CorMatrix (Figure 2). Pathohistological and immunohistochemical analyses confirmed the diagnosis of a high-grade primary intracardiac angiosarcoma. The immunophenotype was vimentin+, CD31+, CD34+, Fil+, SMA+ focal, c-myc+ focal, p53+, MDM2−, Desmin−, S100−, Ki67+ in about 50% of the cells.

Postoperatively, the patient’s inflammatory markers spiked (procalcitonin = 7.44, C-Reactive Protein = 73). Consequently, vancomycin, meropenem, and diflucan were administered. The inflammatory markers eventually returned to the normal range. Due to the decreased hemoglobin levels, two doses of red blood cells were administered. During the hospitalization, the patient was also given low-molecular-weight heparin. Additionally, the patient experienced epistaxis due to a nasal septum injury caused by the nasogastric tube placement, which was controlled with anterior nasal packing. Before discharge, the ECHO revealed pericardial effusion around the right ventricle measuring 25 mm.

The patient was discharged without anticoagulant therapy and with a therapeutic regimen including acetylsalicylic acid, bisoprolol, ramipril, metformin, gliclazide, and pantoprazol. A follow-up was scheduled for one month postoperatively.

**Figure 3:**
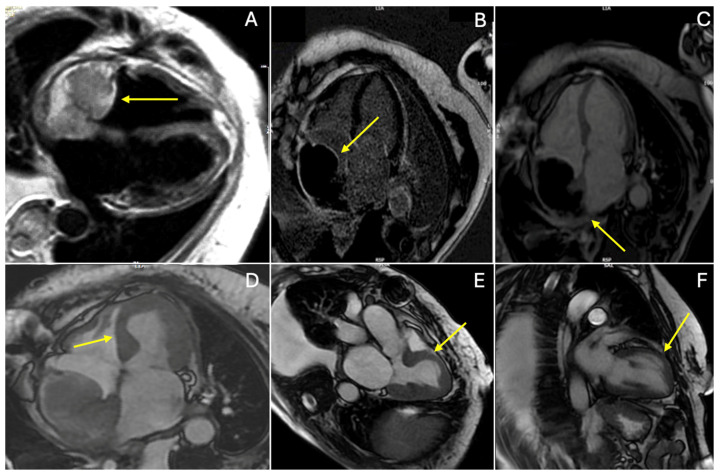
One month follow-up CMR showing RA mass (yellow arrows) appearing in (**A**) black blood T2-weighted image with heterogenous signal; (**B**) late gadolinium enhancement (LGE), showing no contrast uptake in the mass; (**C**) early gadolinium enhancement (EGE), showing no contrast uptake in the mass. Note the hyperintense signal in the black blood T2-weighted image (**A**) in the medial segment of the septum due to edema as part of Takotsubo cardiomyopathy; CMR steady-state free precision cine images in long-axis view showing wall motion abnormalities due to Takotsubo cardiomyopathy (**D**) in four-chamber dyskinesia of the medial segment of septum, (**E**) three-chamber dyskinesia of medial segments of anterior septal wall, and (**F**) two-chamber dyskinesia of medial segments of anterior wall.

At the one-month postoperative visit, the ECHO revealed a large mass occupying the majority of the RA. CMR imaging demonstrated a mass measuring 6.0 × 4.5 cm in the RA, with a heterogeneous T1/T2 signal on black blood images and no contrast uptake on early gadolinium enhancement (EGE) or late gadolinium enhancement (LGE), suggestive of a thrombus (Figure 3). Additionally, dyskinesia of the anterior, septal, medial segments of the left ventricle was noted, with a hyperintense T2 black blood signal without LGE, indicating Takotsubo cardiomyopathy (Figure 3). Disseminated intravascular coagulation was excluded. Given the risks associated with a second surgery within a short period and the presence of Takotsubo cardiomyopathy, the Heart team decided against additional surgical intervention. A 24 h Holter ECG revealed an alternating sinus rhythm with occasional episodes of isorhythmic AV dissociation. A temporary pacemaker was placed, and 7-day telemetric ECG monitoring showed normal findings, with no AV dissociation observed. The patient was prescribed a therapeutic regimen of Warfarin and chemotherapy (Paclitaxel).

**Figure 4:**
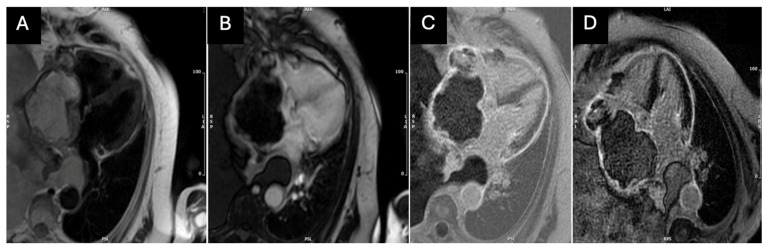
Four-month follow-up CMR imaging showing right-sided mass (**A**) in black blood T2-weighted image long-axis view heterogenous signal; (**B**) early gadolinium enhancement; (**C**) phase-sensitive inversion recovery (PSIR) late gadolinium enhancement (LGE); and (**D**) magnitude IR late gadolinium enhancement (LGE) heterogeneous uptake of contrast.

Four months later, the patient remained dyspnoic (NYHA functional class II). A follow-up CMR showed the normalization of LV function without any wall motion abnormalities and absence of myocardial edema. However, a larger mass was observed in the RA (9.3 × 7.2 cm) with evidence of recurrent angiosarcoma with a superimposed thrombus (heterogeneous T1/T2 black blood signal with post-contrast heterogeneous LGE uptake of the tumor mass) (Figure 4).

## 3. Discussion

The immediate surgical extirpation of the tumor is generally indicated in the early stages of the disease, as demonstrated in our patient [13]. Following the observation of a recurrence of the primary mass at the one-month follow-up, postoperative chemotherapy was initiated, underscoring the critical importance of vigilant monitoring. The literature suggests that postoperative chemotherapy and radiotherapy are beneficial in enhancing local control and reducing recurrence rates [14]. Additionally, extensive cardiac surgery and paraneoplastic syndrome may predispose patients to thrombus formation. Thus, the most appropriate intervention can be chosen, as the literature has shown that survival rates can be improved with an aggressive approach and the use of multiple methods [15,16]. In addition to surgical excision, other management techniques such as microwave ablation have been reported by Filippiadis et al. to achieve bleeding remission in angiosarcoma cases [17]. This technique could be considered as an adjunctive therapy in similar cases to improve outcomes.

Additionally, choosing the appropriate patch for wall repair after extensive cardiac surgery remains a challenge. The CorMatrix patch, a decellularized porcine small intestinal submucosa extracellular matrix, is predominantly used in pediatric heart surgery due to its low probability of immunological response and high potential for growth and re-reendothelialization. Thrombosis has not been described as a complication with this patch. The main histologic complications are inflammatory response, fibrosis of the surrounding tissue, and degeneration of the patch. The selection of a suitable patch is crucial for improving patient outcomes and minimizing postoperative complications [18,19,20].

With regard to anticoagulant therapy, there are no established guidelines defining its necessity after CorMatrix patch reconstruction. During the patient’s hospitalization, low-molecular-weight heparin was administered, but due to the extensive serohemorrhagic pericardial effusion, it was decided to discontinue anticoagulant therapy on discharge, with a recommendation for regular echocardiogram follow-ups to evaluate the pericardial effusion and potential subsequent introduction of anticoagulant therapy.

Furthermore, oncological patients have an increased risk of thromboembolic complications due to the nature of the disease, especially after major surgical procedures. According to the 2022 ESC cardio-oncology guidelines, anticoagulant therapy for cancer patients to prevent venous thromboembolism excludes anticoagulant therapy in patients at a high risk of bleeding (one criterion is pericardial bleeding, as well as transfusion of ≥2 units of red blood cells) and calls for a re-evaluation of thromboembolism and bleeding risks (class I evidence) [21].

While ECHO is a valuable first-line imaging technique, it has inherent limitations, particularly in patients with obesity or poor acoustic windows, which can impede the comprehensive assessment of cardiac and extracardiac structures and soft tissue characteristics [22]. Conversely, CMR imaging offers detailed information regarding mass localization and tissue characteristics, enabling more precise diagnostics and improved patient management [1].

AS typically exhibits a heterogeneous hyperintense appearance on T1- and T2-weighted CMR imaging, attributable to necrosis and hemorrhage within the tumor [23]. First-pass perfusion imaging frequently reveals a “sunray appearance” due to exuberant flow within prominent vascular channels in the tumor [24]. Another distinctive feature of AS on CMR is its cauliflower-like appearance, characterized by nodular areas of increased intensity within regions of low to intermediate signal intensity [11]. The late gadolinium enhancement (LGE) pattern of AS usually shows peripheral rim enhancement with a lack of central enhancement, reflecting central necrosis and hemorrhage [25]. Dynamic steady-state free precession cine sequences are instrumental in assessing kinetic features affected by tumor infiltration, providing insights into tumor mobility, morphology (broad base, lobulation), and its impact on blood flow [26].

An important differential diagnosis to consider is Takotsubo cardiomyopathy, which can occur after a stressful situation like post-cardiac surgery. CMR is particularly advantageous for evaluating this cardiomyopathy, as it facilitates prompt differentiation from acute myocardial infarction and informs immediate conservative medical treatment [27,28].

## 4. Conclusions

The clinical manifestations of angiosarcoma are predominantly dictated by the tumor’s size, location, and degree of invasion. Early-stage diagnosis necessitates prompt surgical intervention to optimize patient outcomes. Adjuvant chemotherapy should be considered for all patients to enhance therapeutic efficacy and reduce recurrence rates. In the presented case, the patient developed a postoperative complication in the form of atrial septal thrombosis. The therapeutic options for this complication include thrombolysis, surgical intervention, and anticoagulant therapy. Given the recent surgical procedure, thrombolysis was contraindicated, and surgical removal posed an increased risk of mortality. Considering the high malignant potential and thrombogenicity of angiosarcoma, the cardiac surgery team decided to manage the thrombus with anticoagulant therapy despite the unfavorable prognosis for thrombus resolution.

The management of postoperative complications in cardiac patients with malignancies such as AS necessitates a meticulous balance between intervention and associated risks. This case illustrates the significance of individualized treatment strategies informed by clinical guidelines and specific patient factors. Early diagnosis and timely surgical intervention are paramount in the treatment of AS. When confronted with complications such as atrial septal thrombosis, anticoagulant therapy may be warranted if alternative interventions are contraindicated. A multidisciplinary approach and comprehensive evaluation of all therapeutic options are essential for optimizing patient outcomes.

## Data Availability

Data are contained within the article.
